# Intractable pneumothorax complicated with interstitial pneumonitis treated with the Tachosuture technique: A case report

**DOI:** 10.1002/ccr3.1782

**Published:** 2018-09-03

**Authors:** Tsuyoshi Uchida, Hirochika Matsubara, Aya Sugimura, Hiroyasu Matsuoka, Tomofumi Ichihara, Hiroyuki Nakajima

**Affiliations:** ^1^ Department of General Thoracic Surgery Yamanashi University Yamanashi Japan

**Keywords:** case reports, interstitial pneumonitis, intractable pneumothorax, Tachosuture technique

## Abstract

It has been proven that the Tachosuture technique is effective for preventing prolonged air leaks caused by pulmonary resection. We successfully used the Tachosuture technique to treat intractable pneumothorax with interstitial pneumonia. This technique avoids pulmonary resection and contributes to acute exacerbations of interstitial pneumonia.

## 1 INTRODUCTION

Interstitial pneumonia (IP) can manifest with secondary spontaneous pneumothorax (SSP).^1^ Treatment of SSP is difficult, because the recurrence rate is high and surgical treatment with pulmonary resection can cause acute exacerbation.^1^, ^2^ Nishida et al reported use of the Tachosuture technique for the prevention of prolonged air leak after pulmonary resection.^3^ The advantage of this treatment method is that it does not require lung resection. We here describe a patient with intractable SSP with IP treated by the Tachosuture technique.

## 2 CASE REPORT

A 76‐year‐old man presented to our department with intractable pneumothorax with IP. Pleurodesis and endobronchial Watanabe spigot embolization were attempted by the previous treating physician after drainage but were ineffective.

**Figure 1:**
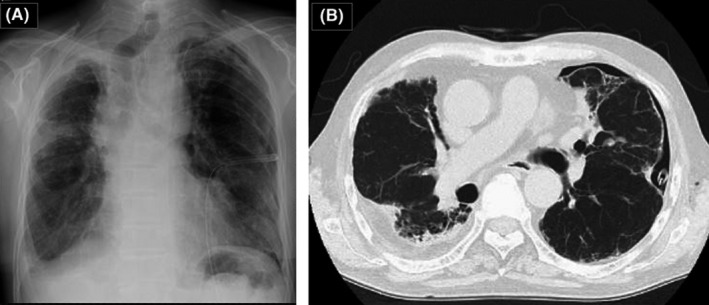
A, There is airway deviation and the lungs are expanded, but overall, the permeability is decreased. The drain position is also inappropriate. (B) Severe fibrotic changes and pleural thickening are observed

The chest x‐ray and computed tomography scan demonstrated severe fibrotic changes in both lung fields, left pneumothorax, and a chest tube that was not appropriately positioned (Figure 1A,B). We prioritized conservative treatment because the patient had been prescribed 15 mg of prednisolone for IP since the age of 74 years. First, the drainage tube was repositioned, and pleurodesis was performed twice. Next, the thoracographic fibrin glue sealing method was performed. However, these treatments failed.

**Figure 2:**
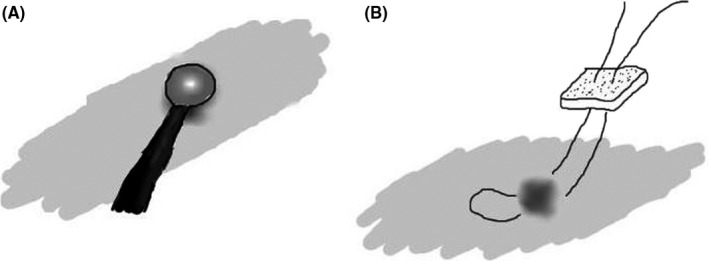
A, The air leakage hole is cauterized with a soft coagulation system. B, Then, the defect is closed with U stitches using 4‐0 Prolene (New Brunswick, New Jersey, USA) and TachoSil (Zurich, Zurich, Switzerland) sutured to the lung surface

We opened the chest through the fifth intercostal space. The apex of the lung was adhered to the chest wall, but there were no other abnormalities identified other than an air leakage point on the dorsal side of the S1+2 segment. The position of the air leak was consistent with the identified region during thoracography. Two bullae were identified with no air leakage. The air leakage defect was cauterized with a soft coagulation system, and then the defect was closed with U stitches using 4‐0 Prolene (Ethicon, New Brunswick, NJ, USA), with TachoSil (Zurich, Zurich, Switzerland) sutured to the lung surface (Figure 2A,B). The other two bullae were covered with TachoSil in the same manner. A chest tube was inserted into the thoracic cavity, and the chest was closed in the typical manner. His postoperative course was favorable.

## 3 DISCUSSION

This case demonstrates two key points: The Tachosuture technique is effective for intractable SSP with IP, and conservative therapy for intractable SSP with IP has limitations.

First, our results show the efficacy of the Tachosuture technique for intractable SSP with IP. This method was reported by Nishida et al^3^ for the prevention of prolonged air leakage after pulmonary resection. TachoSil, a collagen matrix coated with lyophilized human fibrinogen and thrombin, is a surgical sealant that provides a mechanical scaffold for the healing process soon after surgery. The Tachosuture technique is a method of suturing TachoSil, as a patch. With this method, pulmonary resection is not required. In an alternative treatment approach, bulla ligation was reported without pulmonary resection.^4^ In the current case, the lung was very hard; thus, it was difficult to ligate the bullae. Nishida et al^3^ classified pleural defects into three types. They reported that if the pleural defect was large, it can be closed with a TachoSil patch. However, in the current case, it was doubtful whether the same effect could be obtained because the patient's lungs were fibrotic and very hard. We obtained good results using a soft coagulation system to shrink the bullae.

Second, conservative treatment for pneumothorax with IP has limitations. Several reports in the literature state that surgical treatment for pneumothorax with IP has high rates of recurrence and a poor prognosis.^1^, ^2^ For patients who cannot tolerate general anesthesia, treatment with pleurodesis or endobronchial Watanabe spigot embolization is applied. In our patient, a previous treating physician performed endobronchial Watanabe spigot embolization, but this approach failed because the responsible bronchi could not be identified. Pneumothorax with IP carries a risk of acute exacerbation when treated surgically, and the timing of surgery is important because conservative treatment has limited efficacy.

## 4 CONCLUSION

Conservative therapy for SSP with IP can be ineffective, and the Tachosuture technique is a potentially useful approach. It has been previously reported that the Tachosuture technique is effective for preventing prolonged air leakage after pulmonary resection; our case demonstrates that it is an effective treatment option for intractable SSP with IP.

## AUTHORSHIP

HM: drafting the manuscript and revising it. AS: revising the manuscript. HM: revising the manuscript. TI: revising the manuscript. HN: revising the manuscript.

## CONFLICT OF INTEREST

The authors have no conflict of interest to disclose. However, Yamanashi Kousei Hospital paid 2 000 000 yen to the department as a donation.
